# Fishing the Molecular Bases of Treacher Collins Syndrome

**DOI:** 10.1371/journal.pone.0029574

**Published:** 2012-01-25

**Authors:** Andrea M. J. Weiner, Nadia L. Scampoli, Nora B. Calcaterra

**Affiliations:** Instituto de Biología Molecular y Celular de Rosario (IBR), Consejo Nacional de Investigaciones Científicas y Técnicas (CONICET) - Área Biología General, Departamento de Ciencias Biológicas, Facultad de Ciencias Bioquímicas y Farmacéuticas, Universidad Nacional de Rosario, Rosario, Argentina; University of Massachusetts Medical School, United States of America

## Abstract

Treacher Collins syndrome (TCS) is an autosomal dominant disorder of craniofacial development, and mutations in the *TCOF1* gene are responsible for over 90% of TCS cases. The knowledge about the molecular mechanisms responsible for this syndrome is relatively scant, probably due to the difficulty of reproducing the pathology in experimental animals. Zebrafish is an emerging model for human disease studies, and we therefore assessed it as a model for studying TCS. We identified *in silico* the putative zebrafish *TCOF1* ortholog and cloned the corresponding cDNA. The derived polypeptide shares the main structural domains found in mammals and amphibians. *Tcof1* expression is restricted to the anterior-most regions of zebrafish developing embryos, similar to what happens in mouse embryos. *Tcof1* loss-of-function resulted in fish showing phenotypes similar to those observed in TCS patients, and enabled a further characterization of the mechanisms underlying craniofacial malformation. Besides, we initiated the identification of potential molecular targets of treacle in zebrafish. We found that *Tcof1* loss-of-function led to a decrease in the expression of cellular proliferation and craniofacial development. Together, results presented here strongly suggest that it is possible to achieve fish with TCS-like phenotype by knocking down the expression of the *TCOF1* ortholog in zebrafish. This experimental condition may facilitate the study of the disease etiology during embryonic development.

## Introduction

Treacher Collins syndrome, also known as mandibulofacial dysostosis (TCS, OMIM #154500), is an autosomal dominant craniofacial malformation affecting 1∶10,000 newborns. The affected structures have a common embryological origin in the first and second branchial arches. The resulting phenotype is characterized by downslanting palpebral fissures, lower lid coloboma, malar and mandibular hypoplasia, high or cleft palate, external ear malformations, atresia of the hearing canal, and conductive hearing loss [1]. There is striking clinical variability, with the most severe cases leading to perinatal death due to respiratory distress, and mild cases that escape clinical diagnosis. Despite this large spectrum of variability, penetrance is thought to be complete [2].

Mutations in the *Treacher Collins-Franceschetti syndrome 1* gene (*TCOF1*, OMIM *606847), mapped to chromosome 5q32-q33.1, are responsible for over 90% of TCS cases. Over 120 pathogenic mutations have been identified in *TCOF1*. This observation suggests that haploinsufficiency of *TCOF1*'s protein product, treacle, is the underlying cause of TCS [3]. The majority of mutations in *TCOF1* result in truncated treacle proteins [4]–[9], highlighting the importance of the C-terminal domain for treacle function. Treacle appears to participate in ribosome biogenesis by controlling pre-rRNA synthesis or processing [10]–[12]; however, the treacle biological role has not been fully understood yet.

A model of TCS was achieved by knocking-out the mouse *Tcof* gene. Homozygous are lethal and neonatal *Tcof1* ^+/−^ mice die within 24 h of birth [13]. *Tcof1* ^+/−^ mice show fewer mature ribosomes in neuroepithelial and neural crest (NC) cells [13], [14], which were described as the expression territories of *Tcof1* during embryonic development [13], [15]. Recent results show that inhibition of p53 rescues craniofacial abnormalities by preventing apoptotic elimination of NC cells (NCC) [16]. Nevertheless, the ribosome biogenesis was not restored to wild-type levels in rescued mice [16]. This finding suggests that TCS phenotype is not only due to defects in ribosome biogenesis and, moreover, makes imperative to carry out further researches to fully-elucidate the etiology of this pathology.

During the last two decades, the zebrafish has emerged as an important model for vertebrate development as it relates to human health and disease. Several woks using this animal system have provided significant insights into the variety of cellular mechanisms and tissue interactions necessary for proper craniofacial skeleton development [17]. Zebrafish forms essentially all of the same skeletal and muscle tissue types as its higher vertebrate counterparts, but in a simpler pattern, and tissues are composed of fewer cells [18]. In view of this, we have assessed the potentiality of zebrafish for studying the mechanisms responsible for the TCS pathogenesis. Here we report the identification of a zebrafish gene, formerly called B8JIY2 DANRE, as the *TCOF1* ortholog. We cloned and analysed *B8JIY2/tcof1* expression in developing zebrafish and detected a dynamic spatiotemporal pattern similar to that observed for *Tcof1* in mouse. The knockdown of *B8JIY2/tcof1* expression adversely affected the NC development and, furthermore, resulted in fish showing typical features of TCS patients. Remarkably, only craniofacial development was affected since the rest of embryonic structures developed normally in *B8JIY2/tcof1* knocked down zebrafish. Besides, we initiated the identification of potential molecular targets of treacle in zebrafish. Together, results strongly suggest that it is possible to achieve fish with TCS-like phenotype by knocking down the expression of the *TCOF1* orthologous in zebrafish. This experimental condition may facilitate the study of the disease etiology during embryonic development.

## Results

### *In silico* identification of B8JIY2 as the putative ortholog of *TCOF1* in zebrafish

**Figure 1:**
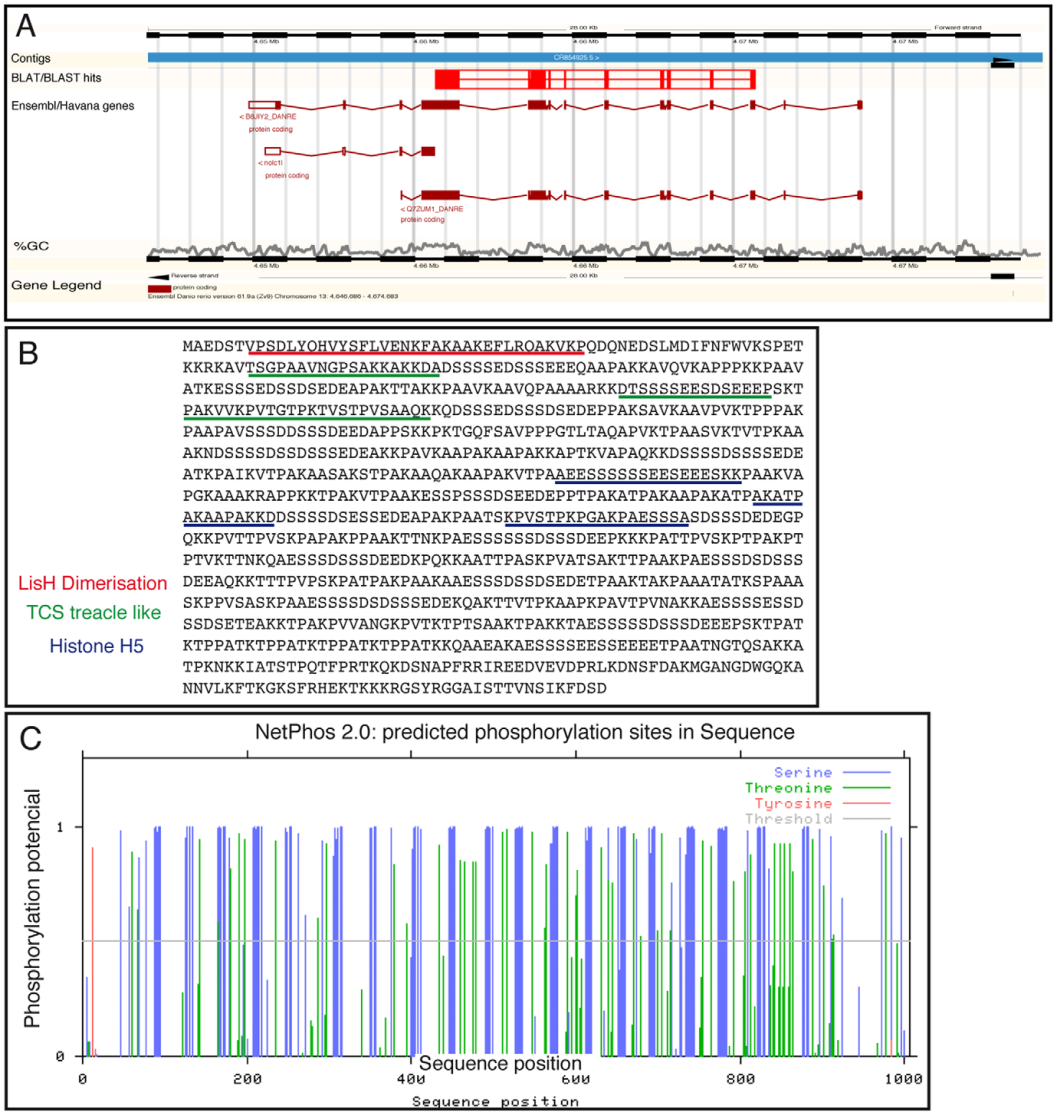
Localization of *B8JIY2* in the zebrafish genome. **A:** *B8JIY2* localize**s** to chromosome 13, spanning the bases 4,646,686 to 4,674,683. The studied contig is represented with a blue rectangle. Blast hits are labeled in red, showing filled boxes for exons and empty boxes for introns. Immediately below, B8JIY2, *nolc1l*, and Q7ZUM1 exons are represented with brown boxes and introns with brown broken lines. Near the bottom, the CG % is shown in grey. **B:** *B8JIY2 in silico* translated amino acid sequence with LisH dimerization (red), TCS treacle like (green), and histone H5 domains (blue) underlined. **C:** NetPhos 2.0 predicted phosphorylation sites in *B8JIY2 in silico* translated amino acid sequence.

There was initially no information related to *tcof1* gene in zebrafish genomic databases. Therefore, we began our analysis using the *tcof1* cDNA sequence from *Xenopus laevis* (GenBank accession number AY731504) to search for homologous sequences in the *Danio rerio* cDNA_ALL Ensembl database (tblastx, Assembly Zv9). Two alignments with a high percentage of identity were located on a region of the minus strand of chromosome 13 (Table S1 and Figure 1A; Chromosome13: 4,655,685–4,665,683). This region of chromosome 13 contains the Ensembl predicted genes B8JIY2_DANRE, Q7ZUM1_DANRE, and *nucleolar and coiled-body phosphoprotein 1-like* or *nolc1l* [19]. Q7ZUM1_DANRE and *nolc1l* sequences partially overlap with the complete region covered by the B8JIY2 sequence (Figure 1A). On the contrary, B8JIY2_DANRE consists of 14 exons that span 19,250 bp in the zebrafish genome (Figure 1A). The predicted protein has 1,001 amino acid residues, a molecular weight of 102,031.33 Da, and an isoelectric point of 9.85. In a similar fashion than in treacle [20], [21], the most relevant features of the *B8JIY2*-derived polypeptide are the presence of an amino terminal LisH dimerisation motif, three TCS treacle-like domains and three histone H5 motifs (Figure 1B). More than 100 putative Ser-phosphorylation sites were predicted using the NetPhos 2.0 application [22], most of which are located within the clusters of serine-acidic residues present within the repeat units (Figure 1C). Finally, because of the high homology observed between mammalian *TCOF1* and *Nolc1* we assessed the percentage of similarity among them and the zebrafish B8JIY2. Data revealed higher similarity of *B8JIY2* with *Tcof1* than with *Nolc1* (Table S2). Collectively, these results strongly suggest that B8JIY2 is the *TCOF1* ortholog in zebrafish. The B8JIY2 sequence was submitted to Gene Bank as the zebrafish *tcof1* gene under submission ID 1430387.

We designed primers to amplify a fragment specific to full-length zebrafish *tcof1* cDNA. RT-PCRs combining different sets of oligonucleotides led to the amplification of two transcripts, which were named *B8JIY2a* and *B8JIY2b*. *In silico* analyses revealed that *B8JIY2b* lacks 103 b of exon 11 (Figure S1). This could be due to either differential pre-mRNA processing or, alternatively, RT-PCR artifacts because the sequence lost in *B8JIY2b* corresponds to a genome repetitive region.

### *Tcof1* is expressed in a dynamic spatiotemporal pattern

**Figure 2:**
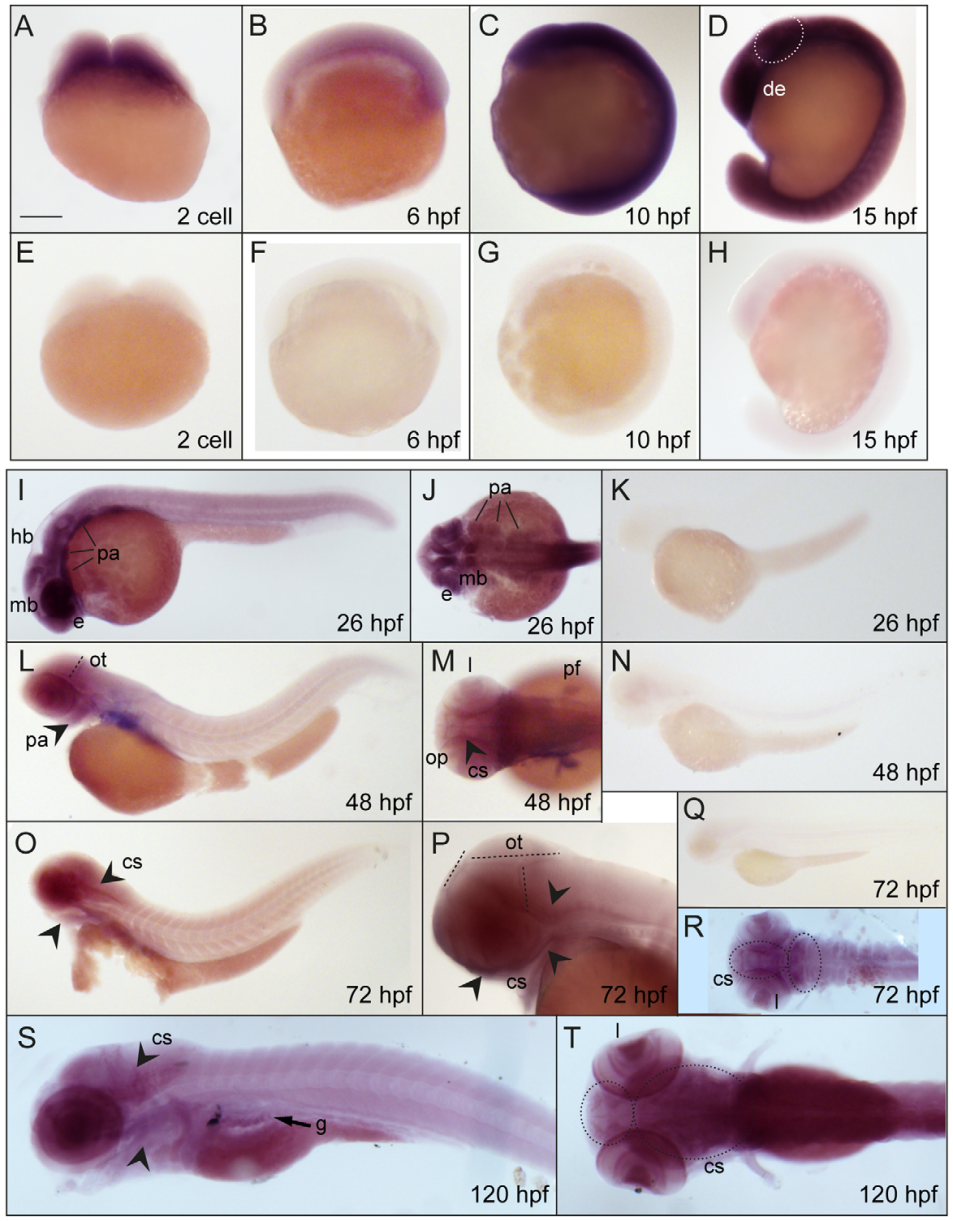
Developmental expression pattern of *tcof1* by whole mount *in situ* hybridization. Lateral (**A**–**I**, **K**, **L**, **N**–**Q**, **S**) and dorsal (**J**, **M**, **R** and **T**) views (anterior regions to the left) of zebrafish embryos hybridized with anti-sense (**A**–**D**, **I**, **J**, **L**, **M**, **O**, **P, and R**–T) or sense (**E**–**H**, **K**, **N** and **Q**) zebrafish *tcof1* probes. The following stages were analyzed: 2-cell stage (**A** and **E**), 6 hpf (**B** and **F**), 10 hpf (**C** and **G**), 13 hpf (**D** and **H**), 24 hpf (**I**–**K**), 48 hpf (**L**–**N**), 72 hpf (**O**–**R**), and 120 hpf (**S**–**T**). Abbreviations: cs, craniofacial structures; de, diencephalic region; e, eye; g, gut; mb, midbrain; hb, hindbrain; l, lenses; op, olfactory pits; ot, optic tectum; pa, pharyngeal arches; pf, pectoral fin buds. Scale bar: 180 µm for **A**–**H**; 210 µm for **I**, **J** and **P**; 266 µm for **K**; 320 µm for **L**, **M** and **R**; 380 µm for **N**, **O**, and **S**–**T**; 530 µm for **Q**.

The following challenge was to analyze the *tcof1* expression pattern. By whole-mount *in situ* hybridization (Figure 2) *Tcof1* mRNA was detected ubiquitously in early staged embryos (Figure 2A–C), indicating a maternally inherited origin. Expression was still ubiquitous in 15 hours post-fertilization (hpf) embryos; however, the optic primordium and prospective brain regions (dotted oval in Figure 2D) showed more intense labeling. At 24 hpf, expression was mainly detected in the prospective eye, midbrain, hindbrain, and the NCC population that later forms a portion of the pre-mandibular, mandibular, first and second branchial arches (Figure 2I–J). At 48 hpf embryos, *tcof1* expression was detected in the retina, pectoral fin buds, pharyngeal arches, prospective craniofacial structures, and olfactory pits (Figure 2L–M). From 48 hpf onwards, the expression observed in brain was refined into a specific pattern along the anterior, dorsal, and posterior edges of the optic tectum (dotted lines in Figure 2L and P). At 72 hpf, *tcof1* expression was observed in the edges of the optic tectum, lenses, and craniofacial structures (arrowheads in Figure 2M, O–P, and dotted oval in R). Later on, 120 hpf-larvae showed expression in the craniofacial structures (arrowheads and dotted circles), lenses, and developing gut (Figure 2S–T). Controls were performed using a *tcof1* sense probe (Figure 2E–H, K, N and Q). Together, results revealed that *tcof1* expression is restricted to the anterior-most regions of zebrafish developing embryos, similar to what happens in mouse embryos [13], [15]. Noteworthy, zebrafish tectal *tcof1* expression profile was highly similar to those of genes involved in cell proliferation, including proto-oncogenes and cell cycle regulatory genes [23]–[26]. This finding is in agreement with previous report showing a role for *Tcof1* in cell proliferation control [16].

**Figure 3:**
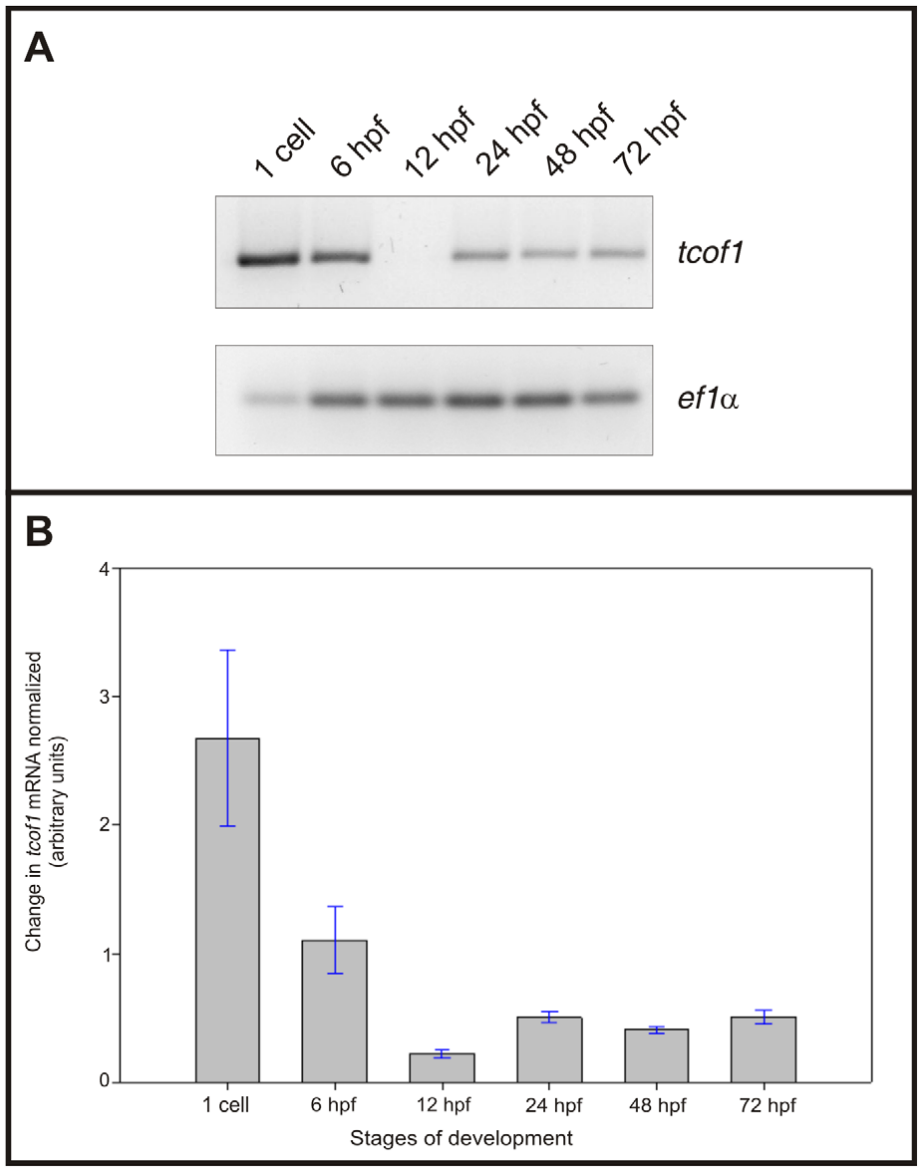
Zebrafish *tcof1* mRNA developmental expression pattern determined by semi-quantitative RT-PCR. **A:** Agarose gel electrophoresis of semi-quantitative RT-PCR products amplified for zebrafish *tcof1* and *ef1α* mRNAs at the 1-cell stage, 6, 12, 24, 48, and 72 hpf stages. **B:** Bar graph of zebrafish *tcof1* mRNA expression profile during embryonic development, normalized to *ef1α* expression (n = 3).

Developmental *tcof1* mRNA distribution was also examined by semi-quantitative RT-PCR (Figure 3). Zebrafish *elongation factor 1 α* (*ef1α*) mRNA was used as a control for RNA quality and for setting linear amplification conditions. Semi-quantitative RT-PCR assays confirmed that *tcof1* is maternally inherited and expressed at high levels during the first stages of zebrafish embryonic development. The highest *tcof1* mRNA expression was observed at the 1-cell stage. Over the course of development, *tcof1* mRNA expression decreased reaching a minimum at 12 hpf. From 12 hpf onwards, *tcof1* expression increased, reaching a plateau at 48 hpf.

Together, results presented here revealed that the *tcof1* spatiotemporal expression pattern is highly dynamic over the course of zebrafish embryonic development. Expression was restricted to regions and developmental stages wherein most of the critical morphogenetic events responsible for craniofacial structure formation take place, including the formation and blending of the branchial arches with the rest of the developing face. Notably, *tcof1* expression decreases once the facial complex has formed, and by 48 hpf onwards, expression becomes reduced near to background levels.

### *Tcof1* loss-of-function results in typical TCS phenotype

TCS patients show mutations in the *TCOF1* gene that result in proteins lacking the C-terminal region [4]–[9]. The loss of the C-terminus leads to mislocalized proteins and consequently to treacle loss-of-function [20], [21]. Importantly, overexpression of C-terminal truncated forms of treacle did not affect the performance of cultured cells, suggesting that TCS results from treacle haploinsufficiency [8], [12]. In view of this, we reasoned that the most informative approach to assess the role of *tcof1* during zebrafish embryonic development would be to decrease *tcof1*-encoded protein levels by generating C-terminal truncated proteins. To achieve this, we designed two morpholino oligonucleotides (MO; [27]) that specifically modify *tcof1* pre-mRNA splicing during embryonic development. One of these MOs, in6:ex7-MO, was directed to the junction between intron 6 and exon 7 while the other one, ex7:in7-MO, was directed to the junction between exon 7 and intron 7. Both MOs targeted splice donor or acceptor sites in *tcof1* exon 7, thereby preventing its inclusion during the pre-mRNA splicing process. *In silico* analysis predicted that the loss of exon 7 results in a premature stop codon, which generates C-terminal truncated treacle forms. This strategy had been successfully used in our laboratory to study the role of other proteins during craniofacial development [28]. Different amounts of MO were injected in 1-cell staged embryos to achieve detectable phenotypes along with the lowest embryonic lethality. Noteworthy, the injection of in6:ex7-MO alone produced higher lethality than the injection of ex7:in7 MO (not shown). The best results were obtained by co-injecting 10.7 ng of each MO per embryo or 21.4 ng of ex7:in7 MO per embryo (Table 1). Lower amount of MO did not result in detectable phenotypes. Controls were performed as described elsewhere [27]. Normal embryos without any phenotype after injection of both the MOs may reflect different genetic backgrounds among fish, similarly to what happens in humans.

**Table 1:** Percentages of normal, affected, or dead embryos/larvae following microinjection with ex7:in7-MO or both MOs at different developmental stages.

		Control	Ex7:in7-MO(21.4 ng)	Both MO(10.7 ng each)
**22 hpf**	Normal (%)	100	4.5	2.9
	TCS-Phenotype (%)	0	42.7	17.4
	Died (%)	0	52.8	79.7
	**TOTAL (** ***n*** **)**	**58**	**89**	**105**
**44 hpf**	Normal (%)	98.3	0	14.3
	TCS-Phenotype (%)	1.7	95.2	71.4
	Died (%)	0	4.8	14.3
	**TOTAL (** ***n*** **)**	**58**	**42**	**21**
**72 hpf**	Normal (%)	98.3	0	16.7
	TCS-Phenotype (%)	0	88.1	72.2
	Died (%)	1.7	11.9	11.1
	**TOTAL (** ***n*** **)**	**58**	**40**	**18**

**Figure 4:**
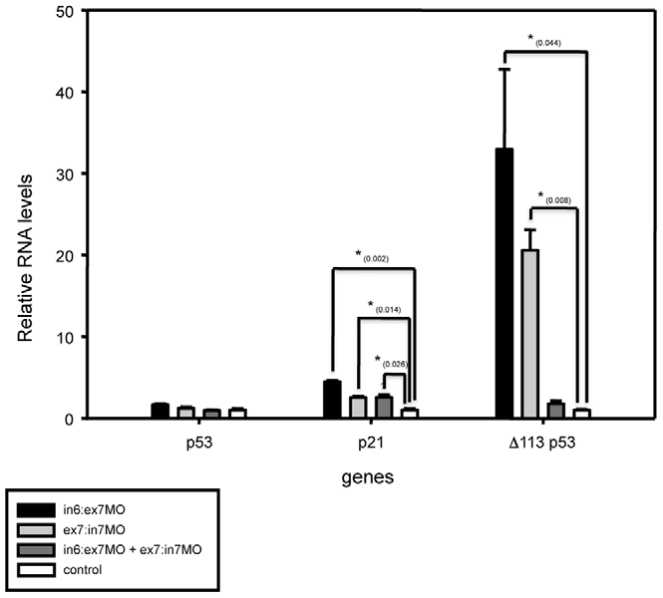
Relative expression levels of putative off-target activated genes in MO-treated embryos using qRT-PCR. The x-axis shows different genes for each sample of MO-treated embryos and the y-axis shows gene expression levels relative to control embryos. Gene expression levels were normalized with *ef1α* and *rpl14* expression. Significant differences are labeled with an asterisk and the p-values are indicated between parentheses for each gene.

Because MO injection may induce neural cell death by off-target activation of p53 and derivatives [29], we checked by real time quantitative RT-PCR (qRT-PCR) the transcriptional levels of p53, p21 and the N-terminal truncated p53 isoform (Δ113 p53) mRNAs in MO-treated embryos at 24 hpf. While full-length p53 RNA levels were not significantly increased in any of the MO-injected zebrafish embryos, the expression of p21 was upregulated in MO-injected embryos and virtually absent in controls (Figure 4). In contrast, the expression of Δ113 p53 isoform, the most reliable marker to diagnostic MO off-target effects [29], was significantly upregulated in in6:ex7-MO- and ex7:in7-MO- injected embryos but did not changed in embryos co-injected with both MOs or in controls (Figure 4). These findings ruled out a MO off-target effect for the latter experimental condition and, therefore, the following experiments were carried out using embryos injected with both MOs at a concentration of 10.7 ng each per embryo. Why a combination of in6:ex7-MO and ex7:in7-MO does not cause MO off-targeting remains completely unknown.

**Figure 5:**
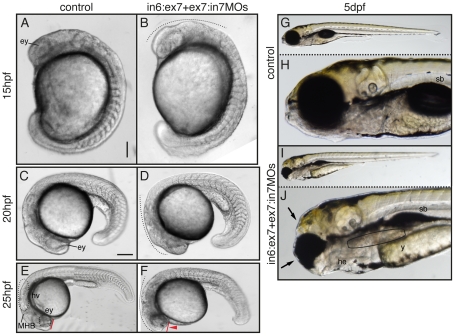
Phenotypic analysis of zebrafish *tcof1* MO-knockdown. Lateral views (anterior to the left) of 15 hpf (**A**–**B**); 20 hpf (**C**–**D**); 25 hpf (**E**–**F**) embryos; and 5 dpf (**G–J**) larvae. Embryos were injected at the 1-cell stage with 1× Danieau (**A**, **C**, **E**, **G** and **H**) or in6:ex7-MO+ex7:in7-MO (**B**, **D**, **F**, **I** and **J**). At 20–25 hpf, the retina and midbrain-hindbrain regions showed a dark pigmentation (dotted lines), suggesting the presence of apoptotic cells. Abbreviations: ey, eye; he, heart edema; hv, hindbrain ventricle; MHB, midbrain-hindbrain border; sb, swim bladder; y, yolk. Scale bar: in **A**, 112.5 µm for **A**–**B**; in **C**, 160 µm for **C**–**D**, 260 µm for **E**–**F**, and 360 µm for **G** and **I**.

Typical morphant phenotypes obtained by *tcof1* loss-of-function are shown in Figure 5. In 15 hpf morphants, the distinctive optic primordium was malformed, the four prominent subdivisions of the brain, the telencephalon, diencephalon, midbrain and hindbrain, as well as the rhombomeres 2 to 6 were undetectable in a dorsolateral view (Figure 5A–B, black dotted line). At 20 hpf, the otic vesicle was barely detectable, and dark apoptotic zones started to appear in the retina and midbrain-hindbrain ventricles (Figure 5C–D, black dotted arch). Morphants at 25 hpf showed eyes reduced in size and opaque apoptotic zones in all brain structures (Figure 5E–F). The typical internal structures seen for the forebrain, midbrain and hindbrain regions in control embryos were severely misarranged in morphants. Besides, morphants showed the dorsal tip of the developing head reduced in extension, being less prominent than in controls (see red lines and arrowhead in Figure 5E–F). At 5 days post-fertilization (dpf), morphant larvae exhibited eyes reduced in size and a shortened and rounded rather than triangular head. Moreover, morphants showed frontonasal hypoplasia manifested by the absence of the typical protruding mouth in front of the eyes (Figure 5G–J). Larvae exhibited heart edema, gut malformations, and swim bladder not fully inflated (see labels in Figure 5J). It should be noted that during all the developmental stages the somites and posterior-most regions were not affected by the knockdown of *tcof1* expression.

We next assessed whether the observed phenotypes were due to the generation of shorter *tcof1* mRNAs analyzing by RT-PCR and cDNA sequencing the pattern and identity of *tcof1* mRNAs present in 15 hpf morphants and controls. RT-PCRs were performed using specific oligonucleotides that hybridized with exons 6 and 8. Specific oligonucleotides for *ef1α* were used to confirm sample quality (not shown). *Tcof1* mRNA variants that lacked exon 7 were detected in MO-injected embryos. The *in silico* translated polypeptide consisted of 235 amino acids that perfectly matched with the first 224 amino acids of the *tcof1*-derived peptide but lacked the C-terminal region (Figure S2). These data strongly suggest that the observed phenotypes result from the reduction of functional protein level due to the generation of a protein isoform that lacks the C-terminal domain.

### *Tcof1* knockdown leads to the reduction of pre-migratory and migratory cranial NCC populations

**Figure 6:**
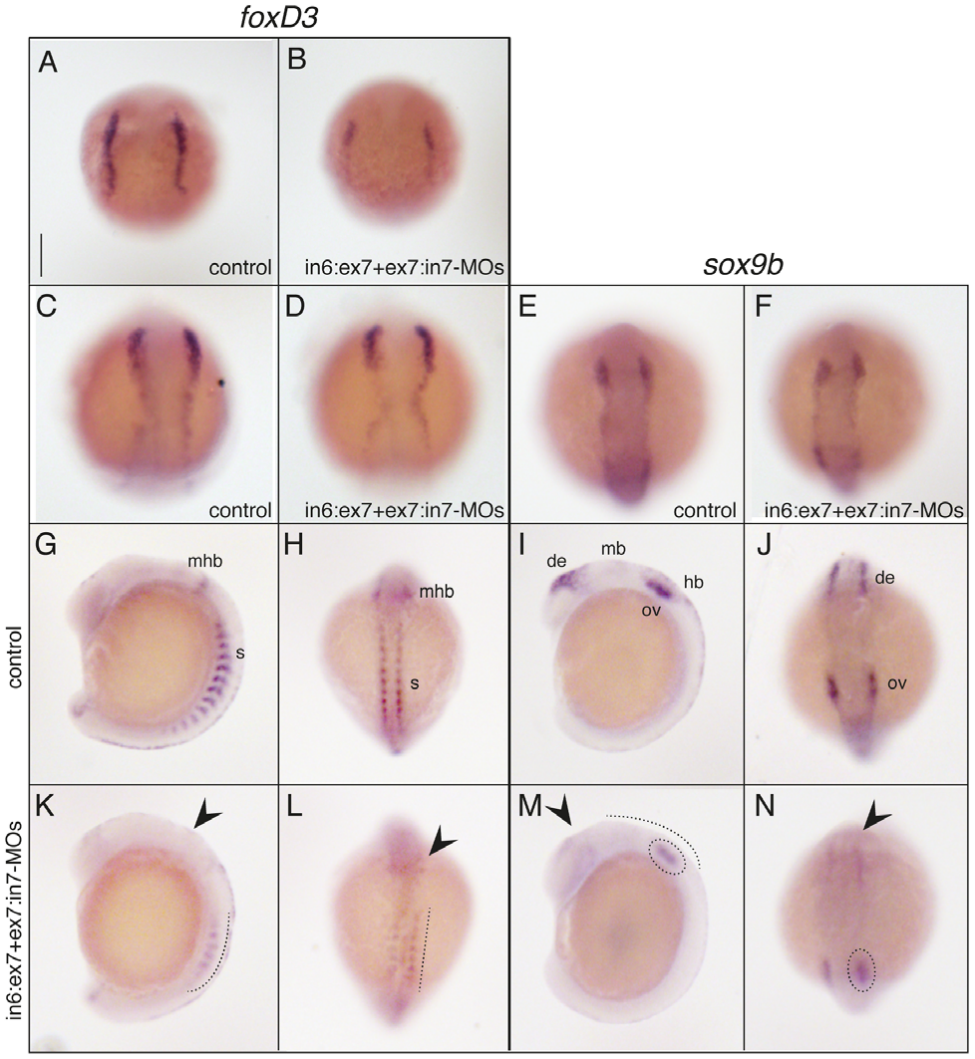
Zebrafish *tcof1* morphants showed reduced NC-specifier gene expression patterns. Lateral (**G**, **I**, **K**, and **M**) and dorsal (**A–F**, **H**, **J**, **L**, and **N**) views of control (**A**, **C**, **E**, and **G**–**J**) and in6:ex7-MO+ex7:in7-MO-treated (**B**, **D**, **F**, **K**–**N**) embryos. Embryos at 14 hpf were analyzed for *foxD3* (**A**–**B** and **E**–**F**) and *sox9b* (**C**–**D** and **G**–**H**) expression patterns using whole-mount *in situ* hybridization. Abbreviations: de, diencephalic; hb, hindbrain; mb, midbrain; mhb, mid-hindbrain; ov, otic vesicles; s, somites. Scale bar: 185 µm for **A**–**N**.

Studies made in *Tcof1* ^+/−^ mice indicate that craniofacial abnormalities observed in TCS patients result from defects in NCC generation, proliferation, and/or viability [13]. Therefore, we employed whole-mount *in situ* hybridization to evaluate the expression patterns of the typical NC-specifier genes *foxd3* and *sox9b* in *tcof1*-knockdown zebrafish embryos (Figure 6). *FoxD3* is one of the earliest NC genes to be expressed in murine, zebrafish, frog, and chick embryos [30], and *sox9b* is involved in iridophores and craniofacial skeleton differentiation [31]. At 10 hpf, *foxD3* expression pattern was clearly reduced in MO-treated embryos compared with controls (Figure 6A–B), although that difference was not so critical at 11 hpf (Figure 6C–D). In 14 hpf morphants, *foxD3* expression was reduced in the somites (see dotted lines in Figure 6K–L) and almost undetectable in the dorsal and anterior brain regions (see arrowheads in Fig. 6K–L, and compare to G–H; 80% of morphant-embryos; n = 25). At 11 hpf, *sox9b* expression pattern was similarly detected in control and MO-treated embryos (Figure 6E–F). In 14 hpf controls, *sox9b* is expressed in NCCs of the diencephalic, midbrain, and hindbrain regions, as well as in the otic vesicle (Figure 6I–J). *Tcof1* knockdown reduced *sox9b* expression, especially in brain regions, in 73% of analyzed embryos (n = 26). Moreover, expression of *sox9b* in the otic vesicles was hardly detected in morphants (Figure 6M–N). These results indicate that the protein encoded by *tcof1* indeed plays a role in the specification and/or establishment of NCCs in zebrafish.

### Craniofacial cartilage formation is adversely affected by *tcof1* knockdown

**Figure 7:**
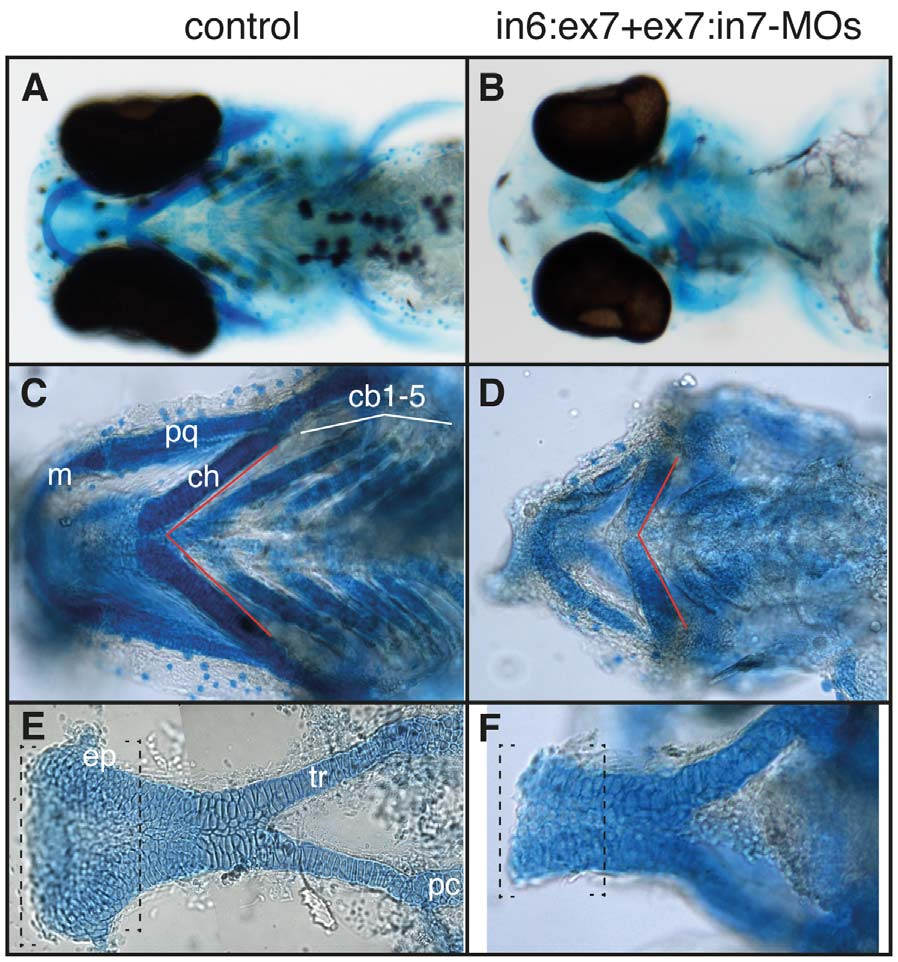
*tcof1* loss-of-function adversely affects zebrafish craniofacial cartilage development. Ventral (**A**–**D**), and dorsal views (**E**–**F**) (anterior to the left) of control (**A**, **C**, and **E**), and in6:ex7-MO+ex7:in7-MO-treated (**B**, **D**, and **F**) 4 dpf larvae stained with Alcian blue. Skeletal staining of control and morphants reveals hypoplasia of numerous craniofacial cartilages. Cranioskeletal hypoplasia is evident in the frontal, premaxillary, and maxillary elements. Abbreviations: cb1–5, ceratobranchial arches 1–5; ch, ceratohyal; ep, ethmoid plate; m, Meckel's cartilage; pc, polar cartilage; pq, palatoquadrate; tr, trabecula.

To extend the study of morphants phenotype, we assessed the consequences of *tcof1* knockdown on zebrafish craniofacial cartilage development. Alcian blue staining revealed that morphant larvae developed reduced pharyngeal arches and neurocranial cartilages (Figure 7A–B). Compared to controls (Figure 7A, C, and E), MO-injected larvae were not able to extend anteriorly the medial edge of Meckel's cartilage or to achieve the typical rounded shape, and ceratobranchial arches 1–5 were difficult to distinguish (Figure 7B, D and F). The ceratohyal cartilage was malformed, and the hyosymplectic and palatoquadrate cartilages, including the pterygoid process, were reduced in size. A variation in the angle formed by ceratohyal cartilages was clearly detected. Compared to controls, this angle was more obtuse in MO-injected fish (Figure 7C–D, red lines). In the neurocranium, the anterior edge of the ethmoid plate appeared smaller and dysmorphic in morphants. The injection of higher concentrations of MOs resulted in the complete loss of either all cartilage elements or Meckel's cartilage (not shown). Further examination at higher magnifications revealed that craniofacial chondrocytes of *tcof1* knocked down larvae have abnormal morphology. In controls, chondrocytes of the ethmoid plate and trabeculae have an ordered organization, with the appearance of a stack of coins (Figure 7E), whereas in *tcof1* morphants, chondrocytes were significantly larger and more rounded in shape (Figure 7F). Taken together, these results demonstrate a phenocopy of the craniofacial abnormalities observed in the *Tcof1* ^+/−^ mouse and TCS patients, including hypoplasia of the jaw cartilages.

### Identification of putative *tcof1* target genes

**Figure 8:**
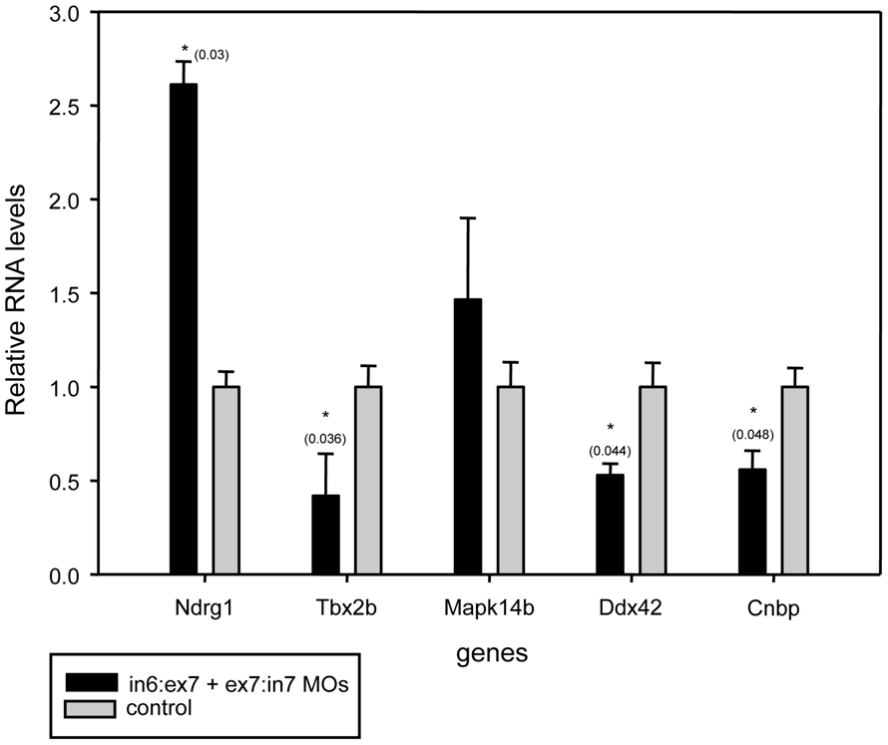
Relative gene expression levels of putative treacle targets using qRT-PCR. The x-axis shows different gene levels analyzed by qRT-PCR performed on RNA samples obtained from either control or MO-treated embryos. The y-axis shows gene expression levels relative to control embryos. Gene expression levels were normalized with *ef1α* and *rpl14* expression. Significant differences are labeled with an asterisk and the p-values are indicated between parentheses for each gene.

By microarray and real time RT-PCR studies, several genes were found to be positively or negatively regulated by *Tcof1* in the mouse NB N1E-115 cell line [32]. Among them, *ndrg1* was up-regulated while *tbx2b* and *cnbp* were down-regulated by *Tcof1* knockdown [32]. Two genes *Mapk14* and *Ddx42* were found to be downregulated with knockdown of *Tcof1* but this was not further confirmed by quantitative real time RT-PCR [32]. In view of this, we wondered if the *tcof1* loss-of-function affects the expression of such target genes in zebrafish developing embryos. The expression of putative *tcof1* targets was assessed by qRT-PCR using total RNA purified from control and *tcof1*-depleted embryos staged at 24 hpf. Specific oligonucleotides were designed for each putative target as indicated in Material and Methods. In agreement with previous results [32], *ndrg1* expression significantly increased, and *tbx2b*, *ddx42* and *cnbp* expression significantly decreased in *tcof1*-depleted embryos (Figure 8). In our experimental conditions, *mapk14b* expression was the only one that did not significantly changed [32].

## Discussion

We report here the identification of the formerly called B8JIY2 sequence as the *TCOF1* ortholog in *Danio rerio*. *Tcof1* and Treacle primary sequences are poorly conserved among mammals and even less conserved among other non-mammalian species [10]. Indeed, human treacle has only 62% amino acid identity with the mouse counterpart protein, and only 19% identity with *Xenopus* treacle [33]. However, several factors strongly support our hypothesis that the cloned cDNA corresponds to the *TCOF1* ortholog in zebrafish, including the LisH domain in the N-terminus; the histone H5 motifs in the C-terminus; its dynamic and anterior-most spatiotemporal expression pattern during embryonic development; its requirement for normal NC and craniofacial cartilage development; and its action on previously informed putative treacle targets. Based on these data, we propose that B8JIY2 is the zebrafish ortholog of the human *TCOF1*.

Cranial cartilages were adversely affected in zebrafish *tcof1* morphants. These cartilages are derived either entirely or in part from NCC and underlying the craniofacial anomalies detected in zebrafish mutants. The number and organization of chondrocytes were abnormal, which may be a consequence of NC aberrant development. *Tcof1* mutant mice show elevated levels of apoptosis within the neural plate, which may initially deplete the neural stem cell pool from which NCC are derived [13]. Therefore, increased apoptosis may eventually reduce the number of migrating cranial NCC, which ultimately results in cranial cartilage hypoplasia. In zebrafish, cranial NCC that originate from the anterior midbrain give rise to the medial ethmoid plate; cranial NCC at the midbrain–hindbrain boundary form the trabecular rods and lateral ethmoid plate; slightly more posterior cranial NCC are involved in the pterygoid process of the palatoquadrate; while even more posterior cranial NCC contribute to the Meckel's cartilage and palatoquadrate [34]–[36]. In our *tcof1*-knockdown morphants, *in situ* hybridization using molecular markers showed pronounced reductions in the pre-migratory and migratory CNC expression domains, comparable to those in *sox9b* mutants (Yan et al, 2005). Therefore, the shorter trabeculae, reduced lateral and medial ethmoid plates and palatoquadrate, as well as the absence of pterygoid processes in *tcof1* morphant are likely to result from a diminution of progenitors by apoptosis of a subset of migratory cranial NCC population.

Experiments carried out in mice have clearly showed that the craniofacial defects displayed by *Tcof1^+/−^* mutants are rescued by inhibiting p53 either genetically or pharmacologically [16]. However, the ribosome biogenesis was still surprisingly impaired in rescued mice. This finding suggests that treacle plays other functions during embryogenesis not related with ribosomal biogenesis or even non-nuclear. In this regard, our data from qRT-PCR opens a new spectrum of possible biological functions for treacle. We found that treacle depletion affects, directly or indirectly, the expression of *ndrg1*, *tbx2*, *ddx42*, and *cnbp* in developing zebrafish embryos. According to this, it seems that expression of *tcof1* as well as those genes that are associated with *tcof1* expression and its protein treacle is required for the proliferation of NCC.

The N-myc downstream-regulated gene 1 (*Ndrg1*) is a metastasis suppressor with anti-tumor function. It reduces metastasis of numerous tumors by up-regulating p21, via p53 independent-mechanisms [37]. Interestingly, a significant increment of p21 expression was observed in MO-treated embryos without affecting p53 expression levels (Figure 4). Furthermore, *Ndrg1* inhibits cancer cell migration [37] and increases apoptosis *in vitro* and *in vivo* [38]. Hence, *tcof1* knockdown may lead to up-regulation of *Ndrg1* which in turn may induce apoptosis of NC precursors, likely by up-regulating the potent cyclin-dependent kinase inhibitor p21.

Transcription factors of the T-box family are required both for early cell-fate decisions, such as those necessary for formation of the basic vertebrate body plan, and for differentiation and organogenesis. In particular Tbx2b mediates cell migration during neural plate formation and neuronal specification in zebrafish [39]. Tbx2 is a known transcriptional repressor and specifically regulates p19ARF in mouse embryo fibroblasts [40]. Activation of p19ARF stabilizes p53 and leads to growth arrest. Therefore *tbx2b* down-regulation in *tcof1* zebrafish morphants may lead to the suppression of p53 function and the concomitant induction of basic apoptotic events during NC formation.

Ddx42 is a putative ATP-dependent RNA helicase implicated in cellular growth and apoptosis. Ddx42 interacts with ASPP2, a major apoptosis inducer known to enhance p53 transactivation of proapoptotic genes. Over-expression of Ddx42 interferes with apoptosis induction by ASPP2, whereas Ddx42 knockdown reduces the survival rate of cultured human cells [41]. Therefore, decreased levels of *ddx42* might promote apoptosis of NCC by failure in ASPP2 controlling.

Finally, *cnbp* has been previously related to both cell proliferation control and craniofacial development. Indeed, *cnbp* codes for a multifunctional nucleic acid chaperone [42], [43] involved in cell death and proliferation control [44], and its knockdown results in aberrant skeletogenic NCC development and severe defects in the pharyngeal skeleton [25], [28], [45]. Besides, *cnbp* developmental expression profile is highly similar to that observed for *tocf1* during zebrafish embryonic development [25], [46]. Collectively, these findings lead us to speculate that loss of Cnbp function goes a long way towards explaining the *tcof1* morphant skeletogenic phenotypes. Therefore, the functional relationship between *tcof1* and *cnbp* and their role in craniofacial development will certainly be essential topics for further studies.

This work was designed to introduce the scientific community to the possibilities associated with studying TCS in zebrafish. Our principal findings include the establishment of TCS model in zebrafish, and the identification of a set of genes as putative candidates for explaining TCS symptoms and designing therapeutic prevention strategies for TCS. The studies we describe here are limited in scope, but it is our belief that further and more sophisticated use of this simple vertebrate species could lead to shed new light on potential therapeutic avenues for the prevention not only of TCS but also of other congenital craniofacial disorders which may share a similar etiology and pathogenesis.

## Materials and Methods

### Fish and embryo rearing

Adult zebrafish were maintained at 28°C on a 14-h light/10-h dark cycle as previously described (Westerfield et al 1995). All embryos were staged according to development in hpf or dpf at 28°C (Kimmel et al. 1995), as well as handled in compliance with relevant national and international guidelines. Protocols were approved by the Commission of Bioethics, Facultad de Cs. Bioquímicas y Farmacéuticas-UNR, which has been accepted by the Ministerio de Salud de la Nación Argentina (http://www.saludinvestiga.org.ar/comites.asp?num_prov=13).

### RNA extraction and RT-PCR analysis

Total RNA from embryos at different developmental stages was obtained using TRIZOL® Reagent (Invitrogen) following the manufacturer's instructions. Purified RNA was incubated with RQ1 DNAse (Promega) and used to perform RT-PCR. Total RNA was retro-transcribed with SuperScript II enzyme (Invitrogen) and either poly-dT or sequence-specific oligonucleotides. For B8JIY2 cDNA cloning, combinations of seven forward primers and six reverse primers were employed. The sequences of the oligonucleotides were: Fwd1_ B8JIY2 (AGCCACGTCCAAACCAGTG), Fwd2_ B8JIY2 (TCAGACGAGGAACCGAAAAAG), Fwd3_ B8JIY2 (CTCAGAAGAAGCAGGACAGCAG), Fwd4_ B8JIY2 (CTCCGATGAGGAAGATGCTC), Fwd5_ B8JIY2 (GTACTCGTTTCTCGTGGAAAACA), Fwd6_ B8JIY2 (CAGGACAGCACGGTTCCTA), Fwd7_ B8JIY2 (GGCGGAGGAATCTTCATCTT), Rvs1_ B8JIY2 (GGTGTTTTAGACGGCTCCTCTTC), Rvs2_ B8JIY2 (CTGAACTACTGCTCTCCGCTTTC), Rvs3_ B8JIY2 (TAGGAGTACTCACTGGTTTGGAC), Rvs4_ B8JIY2 (CTGTCCGAGGCTGAGCTAGA), Rvs5_ B8JIY2 (ATCACTAGAGCTGTCCTCACTGC), Rvs6_ B8JIY2 (GACTGGAGCTTGAGCCGTCAG). The isoforms generated by MO treatment were analyzed with a specific B8JIY2 exon 6 forward primer (CTCTAGAAGAAGCAGGACAGCAG) and an exon 8 reverse primer (GACTGGAGCTTGAGCCGTCAG). Products were resolved in 1.2% (w/v) agarose gels stained with Gel Green, cloned into pGEM-T Easy Vector System (Promega), and their sequences obtained by University of Maine DNA Sequencing Facility. *Ef1α* RT-PCR was performed as a control and for setting conditions [47], which were established at 24 amplification cycles. For qRT-PCR, first strand cDNA was reverse-transcribed from total RNA purified from 24 hpf embryo as described above and using oligo(dT) primer. Reactions were performed using four different RNA purifications and three independent experiments using an Eppendorf realplex2 and standard temperature protocol were performed. Primer sequences for *ef1α*, *rpl14*, *p53*, *p21* and *Δ113 p53* were used as described elsewhere [29]. The resting primer sequences were obtained using Primer3 program, as follows: *Ndrg1f*, GACCAAAACCACACTGCTAAAGAT; *Ndrg1r*, GAATGAAGTACTTGAAAGCCTCTG; *Tbx2bf*, CTTTCATCTGTCTCAACACATGCT; *Tbx2br*, GTGTAGGGGTACGGGAAAAGTC; *Mapk14bf*, CTCATCCGTATTTCGCTCAGTATC; *Mapk14br*, ATACGTCAGACTTTTCCACTCCTC; *Ddx42f*, AGCTCTTGGCAACTATGAAACCT; *Ddx42r*, CGACAAAGTGGTTTTTGTACTGAG. Expected PCR products span at least one intron (except the *Δ113 p53* fragment), to ensure amplification solely from the cDNA and not from genomic DNA. *Ef1α* and *rpl14* were used as endogenous control for normalization analysis. Acquired data were analyzed using qBase software version 2.2, and p-values were obtained from *t*-student analysis.

### Sequences analysis

cDNAs were sequenced by the DNA Sequencing Facility of University of Maine. Complete mRNA and gene organization analysis were obtained from the www.ensembl.org website. Sequence alignments and similarity indexes calculations were carried out using ClustalW2 version 2.0.12 (http://www.ebi.ac.uk/Tools/msa/clustalw2/). The B8JIY2 sequence was submitted to Gene Bank as the zebrafish *tcof1* gene under submission ID 1430387.

### mRNA *in situ* hybridization

Embryos were staged and fixed overnight in 4% (w/v) paraformaldehyde (PFA) in 1× PBS at 4°C. After washing, embryos were stored in methanol at −20°C until use. Whole-mount *in situ* hybridization was carried out as previously described [48]. Digoxigenin-UTP-labeled sense and anti-sense riboprobes were prepared according to the manufacturer's instructions (Roche Diagnostics, Mannheim). B8JIY2 exon 11 was used as the detection probe. *FoxD3 and sox9b* expression patterns were analyzed as described elsewhere [25].

### Antisense morpholino oligonucleotides

Embryos were obtained by natural mating and injected at the one-cell stage into the yolk immediately below the blastomeres using a gas-driven microinjection apparatus (MPPI-2 Pressure Injector, Applied scientific Instrumentation; Eugene, OR, USA). MOs were designed and synthesized by Gene Tools (Philomath, OR, USA). The in6:ex7-MO sequence is: 5′-CGCTCTGTGAGAAGAGTAAGAATAT-3′, and the ex7:in7-MO sequence is: 5′-ATGAGAAAGTTAGGCAATACCAGTC-3′. Embryos were injected with 5 nl of the optimum concentration of MO prepared in 1× Danieau [27].

### Alcian Blue staining

Four dpf larvae were fixed in 4% (w/v) PFA in 1× PBT, washed in 1× PBT four times, and treated as described elsewhere [49].

## Supporting Information

Figure S1 **Multiple sequence alignment of B8JIY2a, B8JIY2b, and B8JIY2 using Clustal 2.0.12.** B8JIY2a and B8JIY2b sequences correspond to the two amplified, cloned and sequenced versions while B8JIY2 was obtained from Ensembl database. The alignment shows the deleted region presented in B8JIY2b sequence at around 1548 bases.
(TIF)

Figure S2 **Sequence alignment of tcof1 and tcof1Δex7 using Clustal 2.0.12.** The alignment highlights that the protein translated from the tcof1exon7-deleted version, which is generated in embryos injected with specific Morpholinos, do not have the three Histone H5 C-terminal motifs.
(TIF)

Table S1 **Results from the cDNA_ALL database of Ensembl (tblastx) search using the** ***tcof1 Xenopus laevis*** **cDNA sequence as query.**
(DOC)

Table S2 **Similarity indexes for the alignment of** ***Tcof1*** **and** ***Nolc1*** **sequences from** ***H. sapiens*** **,** ***R. norvegicus*** **,** ***M. musculus*** **and** ***X. laevis*** **, and B8JIY2 and Q7ZUM1 from** ***D. rerio*** **.** Sequence alignments and similarity indexes calculations were carried out using the software package GeneDoc ver. 2.6. (www.psc.edu/biomed/genedoc).
(DOC)

## References

[1] Passos-Bueno MR, Ornelas CC, Fanganiello RD (2009). Syndromes of the first and second pharyngeal arches: A review.. Am J Med Genet A.

[2] Dixon J, Dixon MJ (2004). Genetic background has a major effect on the penetrance and severity of craniofacial defects in mice heterozygous for the gene encoding the nucleolar protein Treacle.. Dev Dyn.

[3] Splendore A, Fanganiello RD, Masotti C, Morganti LS, Passos-Bueno MR (2005). TCOF1 mutation database: novel mutation in the alternatively spliced exon 6A and update in mutation nomenclature.. Hum Mutat.

[4] Splendore A, Jabs EW, Felix TM, Passos-Bueno MR (2003). Parental origin of mutations in sporadic cases of Treacher Collins syndrome.. Eur J Hum Genet.

[5] Wise CA, Chiang LC, Paznekas WA, Sharma M, Musy MM (1997). TCOF1 gene encodes a putative nucleolar phosphoprotein that exhibits mutations in Treacher Collins Syndrome throughout its coding region.. Proc Natl Acad Sci U S A.

[6] Marszalek B, Wisniewski SA, Wojcicki P, Kobus K, Trzeciak WH (2003). Novel mutation in the 5′ splice site of exon 4 of the TCOF1 gene in the patient with Treacher Collins syndrome.. Am J Med Genet A.

[7] Edwards SJ, Gladwin AJ, Dixon MJ (1997). The mutational spectrum in Treacher Collins syndrome reveals a predominance of mutations that create a premature-termination codon.. Am J Hum Genet.

[8] Splendore A, Jabs EW, Passos-Bueno MR (2002). Screening of TCOF1 in patients from different populations: confirmation of mutational hot spots and identification of a novel missense mutation that suggests an important functional domain in the protein treacle.. J Med Genet.

[9] Splendore A, Silva EO, Alonso LG, Richieri-Costa A, Alonso N (2000). High mutation detection rate in TCOF1 among Treacher Collins syndrome patients reveals clustering of mutations and 16 novel pathogenic changes.. Hum Mutat.

[10] Gonzales B, Yang H, Henning D, Valdez BC (2005). Cloning and functional characterization of the Xenopus orthologue of the Treacher Collins syndrome (TCOF1) gene product.. Gene.

[11] Hayano T, Yanagida M, Yamauchi Y, Shinkawa T, Isobe T (2003). Proteomic analysis of human Nop56p-associated pre-ribosomal ribonucleoprotein complexes. Possible link between Nop56p and the nucleolar protein treacle responsible for Treacher Collins syndrome.. J Biol Chem.

[12] Valdez BC, Henning D, So RB, Dixon J, Dixon MJ (2004). The Treacher Collins syndrome (TCOF1) gene product is involved in ribosomal DNA gene transcription by interacting with upstream binding factor.. Proc Natl Acad Sci U S A.

[13] Dixon J, Jones NC, Sandell LL, Jayasinghe SM, Crane J (2006). Tcof1/Treacle is required for neural crest cell formation and proliferation deficiencies that cause craniofacial abnormalities.. Proc Natl Acad Sci U S A.

[14] Dixon J, Brakebusch C, Fassler R, Dixon MJ (2000). Increased levels of apoptosis in the prefusion neural folds underlie the craniofacial disorder, Treacher Collins syndrome.. Hum Mol Genet.

[15] Dixon J, Hovanes K, Shiang R, Dixon MJ (1997). Sequence analysis, identification of evolutionary conserved motifs and expression analysis of murine tcof1 provide further evidence for a potential function for the gene and its human homologue,. TCOF1 Hum Mol Genet.

[16] Jones NC, Lynn ML, Gaudenz K, Sakai D, Aoto K (2008). Prevention of the neurocristopathy Treacher Collins syndrome through inhibition of p53 function.. Nat Med.

[17] Parsons KJ, Andreeva V, James CW, Yelick PC, Craig AR (2011). Morphogenesis of the zebrafish jaw: development beyond the embryo.. Methods Cell Biol.

[18] Schilling TF, Kimmel CB (1997). Musculoskeletal patterning in the pharyngeal segments of the zebrafish embryo.. Development.

[19] Hubbard T, Barker D, Birney E, Cameron G, Chen Y (2002). The Ensembl genome database project.. Nucleic Acids Res.

[20] Marsh KL, Dixon J, Dixon MJ (1998). Mutations in the Treacher Collins syndrome gene lead to mislocalization of the nucleolar protein treacle.. Hum Mol Genet.

[21] Winokur ST, Shiang R (1998). The Treacher Collins syndrome (TCOF1) gene product, treacle, is targeted to the nucleolus by signals in its C-terminus.. Hum Mol Genet.

[22] Blom N, Gammeltoft S, Brunak S (1999). Sequence and structure-based prediction of eukaryotic protein phosphorylation sites.. J Mol Biol.

[23] Duffy KT, McAleer MF, Davidson WR, Kari L, Kari C (2005). Coordinate control of cell cycle regulatory genes in zebrafish development tested by cyclin D1 knockdown with morpholino phosphorodiamidates and hydroxyprolyl-phosphono peptide nucleic acids.. Nucleic Acids Res.

[24] Loeb-Hennard C, Kremmer E, Bally-Cuif L (2005). Prominent transcription of zebrafish N-myc (nmyc1) in tectal and retinal growth zones during embryonic and early larval development.. Gene Expr Patterns.

[25] Weiner AM, Allende ML, Becker TS, Calcaterra NB (2007). CNBP mediates neural crest cell expansion by controlling cell proliferation and cell survival during rostral head development.. J Cell Biochem.

[26] Wullimann MF, Knipp S (2000). Proliferation pattern changes in the zebrafish brain from embryonic through early postembryonic stages.. Anat Embryol (Berl).

[27] Nasevicius A, Ekker SC (2000). Effective targeted gene ‘knockdown’ in zebrafish.. Nat Genet.

[28] Weiner AM, Sdrigotti MA, Kelsh RN, Calcaterra NB (2011). Deciphering the cellular and molecular roles of CNBP during cranial neural crest development.. Dev Growth Differ.

[29] Robu ME, Larson JD, Nasevicius A, Beiraghi S, Brenner C (2007). p53 activation by knockdown technologies.. PLoS Genet.

[30] Steventon B, Carmona-Fontaine C, Mayor R (2005). Genetic network during neural crest induction: from cell specification to cell survival.. Semin Cell Dev Biol.

[31] Yan YL, Willoughby J, Liu D, Crump JG, Wilson C (2005). A pair of Sox: distinct and overlapping functions of zebrafish sox9 co-orthologs in craniofacial and pectoral fin development.. Development.

[32] Mogass M, York TP, Li L, Rujirabanjerd S, Shiang R (2004). Genomewide analysis of gene expression associated with Tcof1 in mouse neuroblastoma.. Biochem Biophys Res Commun.

[33] Walker MB, Trainor PA (2006). Craniofacial malformations: intrinsic vs extrinsic neural crest cell defects in Treacher Collins and 22q11 deletion syndromes.. Clin Genet.

[34] Eberhart JK, Swartz ME, Crump JG, Kimmel CB (2006). Early Hedgehog signaling from neural to oral epithelium organizes anterior craniofacial development.. Development.

[35] Kimmel CB, Miller CT, Moens CB (2001). Specification and morphogenesis of the zebrafish larval head skeleton.. Dev Biol.

[36] Wada N, Javidan Y, Nelson S, Carney TJ, Kelsh RN (2005). Hedgehog signaling is required for cranial neural crest morphogenesis and chondrogenesis at the midline in the zebrafish skull.. Development.

[37] Kovacevic Z, Sivagurunathan S, Mangs H, Chikhani S, Zhang D (2011). The metastasis suppressor, N-myc downstream regulated gene 1 (NDRG1), upregulates p21 via p53-independent mechanisms.. Carcinogenesis.

[38] Angst E, Dawson DW, Stroka D, Gloor B, Park J (2011). N-myc downstream regulated gene-1 expression correlates with reduced pancreatic cancer growth and increased apoptosis in vitro and in vivo.. Surgery.

[39] Fong SH, Emelyanov A, Teh C, Korzh V (2005). Wnt signalling mediated by Tbx2b regulates cell migration during formation of the neural plate.. Development.

[40] Jacobs JJ, Keblusek P, Robanus-Maandag E, Kristel P, Lingbeek M (2000). Senescence bypass screen identifies TBX2, which represses Cdkn2a (p19(ARF)) and is amplified in a subset of human breast cancers.. Nat Genet.

[41] Uhlmann-Schiffler H, Kiermayer S, Stahl H (2009). The DEAD box protein Ddx42p modulates the function of ASPP2, a stimulator of apoptosis.. Oncogene.

[42] Armas P, Nasif S, Calcaterra NB (2008). Cellular nucleic acid binding protein binds G-rich single-stranded nucleic acids and may function as a nucleic acid chaperone.. J Cell Biochem.

[43] Borgognone M, Armas P, Calcaterra NB (2010). Cellular nucleic-acid-binding protein, a transcriptional enhancer of c-Myc, promotes the formation of parallel G-quadruplexes.. Biochem J.

[44] Calcaterra NB, Armas P, Weiner AM, Borgognone M (2010). CNBP: A multifunctional nucleic acid chaperone involved in cell death and proliferation control.. IUBMB Life.

[45] Armas P, Aguero TH, Borgognone M, Aybar MJ, Calcaterra NB (2008). Dissecting CNBP, a zinc-finger protein required for neural crest development, in its structural and functional domains.. J Mol Biol.

[46] Armas P, Cachero S, Lombardo VA, Weiner A, Allende ML (2004). Zebrafish cellular nucleic acid-binding protein: gene structure and developmental behaviour.. Gene.

[47] Tang R, Dodd A, Lai D, McNabb WC, Love DR (2007). Validation of zebrafish (Danio rerio) reference genes for quantitative real-time RT-PCR normalization.. Acta Biochim Biophys Sin (Shanghai).

[48] Jowett T, Lettice L (1994). Whole-mount in situ hybridizations on zebrafish embryos using a mixture of digoxigenin- and fluorescein-labelled probes.. Trends Genet.

[49] Solomon KS, Kudoh T, Dawid IB, Fritz A (2003). Zebrafish foxi1 mediates otic placode formation and jaw development.. Development.

