# Supplementary figures and images for: Fishing the Molecular Bases of Treacher Collins Syndrome
Source: PLoS One. 2012 Jan 25;7(1):e29574. doi: 10.1371/journal.pone.0029574 (PMC3266255; doi:10.1371/journal.pone.0029574)

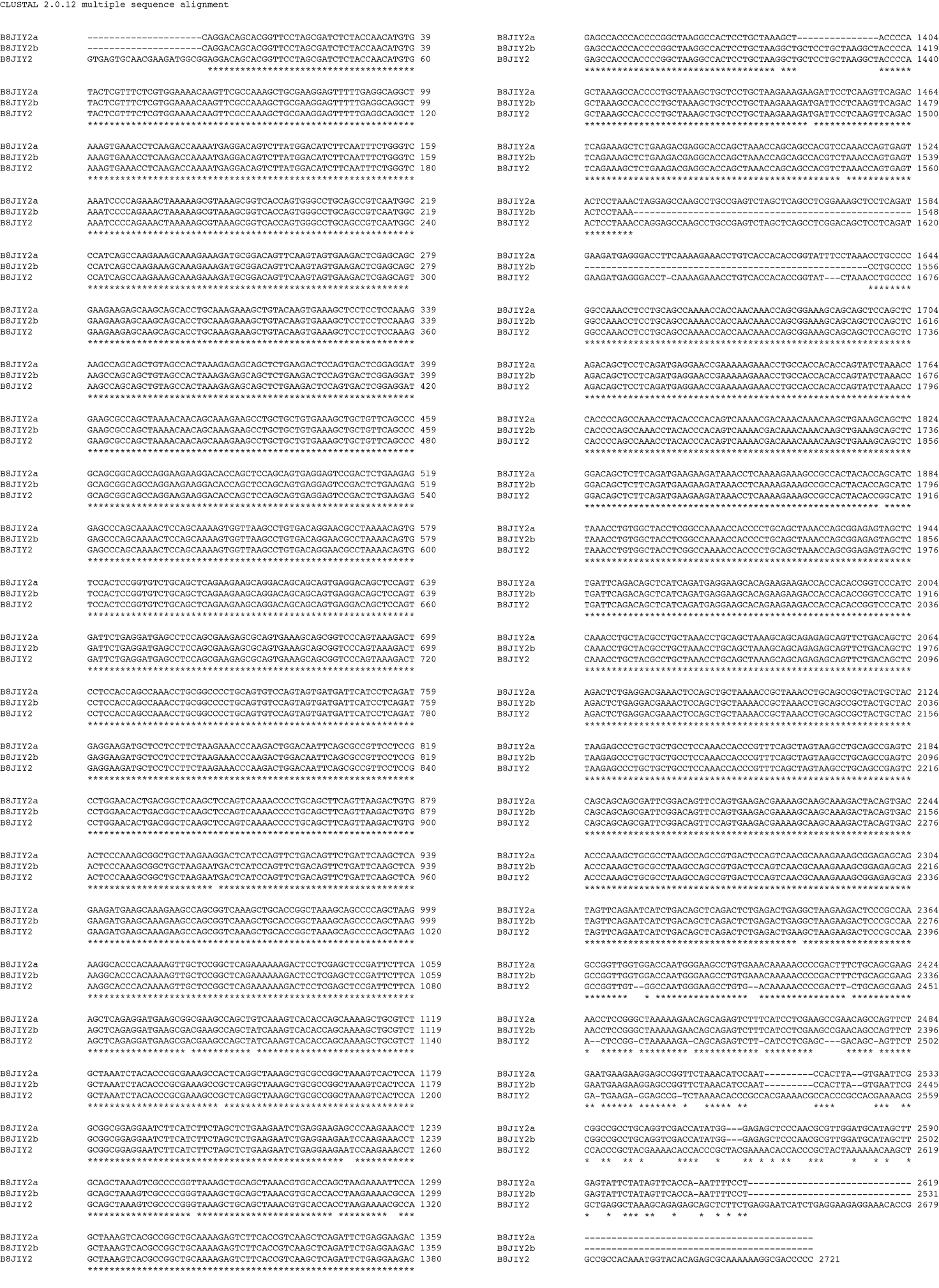

Supplement: Figure S1 — Multiple sequence alignment of B8JIY2a, B8JIY2b, and B8JIY2 using Clustal 2.0.12. B8JIY2a and B8JIY2b sequences correspond to the two amplified, cloned and sequenced versions while B8JIY2 was obtained from Ensembl database. The alignment shows the deleted region presented in B8JIY2b sequence at around 1548 bases. (TIF) [file pone.0029574.s001.tif]

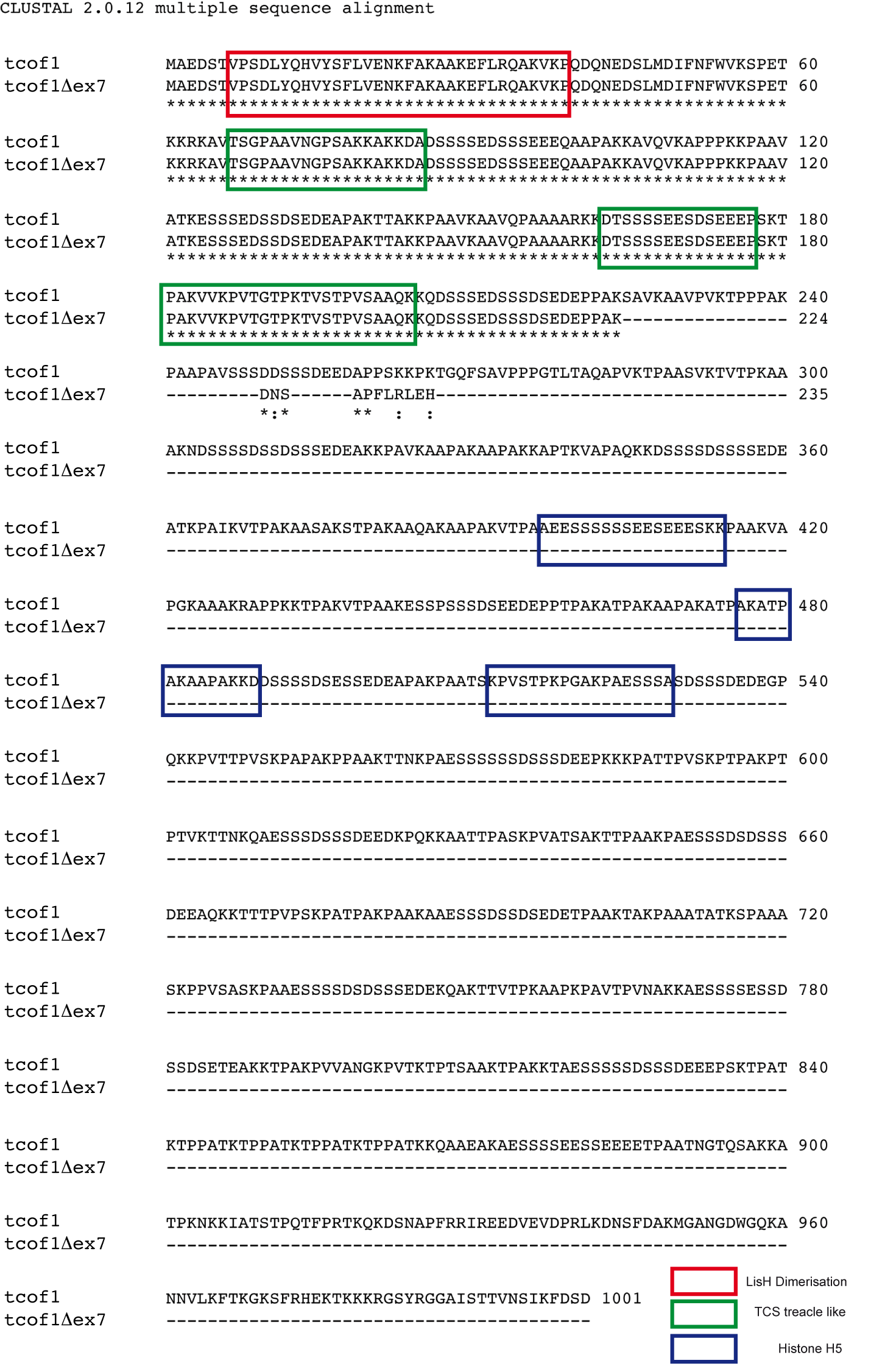

Supplement: Figure S2 — Sequence alignment of tcof1 and tcof1Δex7 using Clustal 2.0.12. The alignment highlights that the protein translated from the tcof1exon7-deleted version, which is generated in embryos injected with specific Morpholinos, do not have the three Histone H5 C-terminal motifs. (TIF) [file pone.0029574.s002.tif]
