# Supplementary material for: Fishing the Molecular Bases of Treacher Collins Syndrome
Source: PLoS One. 2012 Jan 25;7(1):e29574. doi: 10.1371/journal.pone.0029574 (PMC3266255; doi:10.1371/journal.pone.0029574)
Supplement: Table S1 — Results from the cDNA_ALL database of Ensembl (tblastx) search using the tcof1 Xenopus laevis cDNA sequence as query. (DOC) [file pone.0029574.s003.doc]

**Supplementary Table S1:** Results from the cDNA_ALL database of Ensembl (tblastx) search using the *tcof1* *Xenopus laevis* cDNA sequence as query.

| Query | | Subject | | | | Chromosome | | | | Stats | | | | |
| --- | --- | --- | --- | --- | --- | --- | --- | --- | --- | --- | --- | --- | --- | --- |
| START | END | Ori | Name | START | END | Ori | Name | START | END | Ori | Score | E-val | %ID | Length |
| 1960 | 4017 | + | ENSDART00000127465 | 193 | 2238 | + | Chr13 | 4655685 | 4665683 | - | 146 | 1.30E-05 | 21.17 | 718 |
| 1960 | 4017 | + | ENSDART00000102651 | 193 | 2253 | + | Chr13 | 4655685 | 4665683 | - | 140 | 6.30E-05 | 19.75 | 709 |
| 950 | 1993 | + | ENSDART00000127194 | 566 | 1588 | + | Chr8 | 2363940 | 2364962 | + | 139 | 8.50E-05 | 27.2 | 375 |
| 1469 | 3091 | + | ENSDART00000127294 | 53 | 1591 | + | Chr8 | 2360758 | 2364965 | + | 138 | 0.00011 | 24.69 | 559 |
| 1532 | 2794 | + | ENSDART00000123827 | 281 | 1528 | + | Chr2 | 47441943 | 47443190 | + | 121 | 0.0078 | 25.55 | 458 |
