# Supplementary material for: Fishing the Molecular Bases of Treacher Collins Syndrome
Source: PLoS One. 2012 Jan 25;7(1):e29574. doi: 10.1371/journal.pone.0029574 (PMC3266255; doi:10.1371/journal.pone.0029574)
Supplement: Table S2 — Similarity indexes for the alignment of Tcof1 and Nolc1 sequences from H. sapiens , R. norvegicus , M. musculus and X. laevis , and B8JIY2 and Q7ZUM1 from D. rerio . Sequence alignments and similarity indexes calculations were carried out using the software package GeneDoc ver. 2.6. (www.psc.edu/biomed/genedoc). (DOC) [file pone.0029574.s004.doc]

Supplementary Table S2: Similarity indexes for the alignment of *Tcof1* and *Nolc1* sequences from *H. sapiens*, *R. norvegicus*, *M. musculus* and *X. laevis,* and B8JIY2 and Q7ZUM1 from *D. rerio*.

| *TCOF1* | *H. sapiens* | *R. norvegicus* | *M. musculus* | *X. laevis* | B8JIY2 | Q7ZUM1 |
| --- | --- | --- | --- | --- | --- | --- |
| *H. sapiens* | - | 73 | 72 | 56 | 65 | 68 |
| *R. norvegicus* | 73 | - | 87 | 53 | 63 | 67 |
| *M. musculus* | 72 | 87 | - | 52 | 64 | 66 |
| *X. laevis* | 56 | 53 | 52 | - | 65 | 67 |
| B8JIY2 | 65 | 63 | 64 | 65 | - | 100 |
| Q7ZUM1 | 68 | 67 | 66 | 67 | 100 | - |
|  |  |  |  |  |  |  |
|  |  |  |  |  |  |  |
|  |  |  |  |  |  |  |
| *NOLC1* | *H. sapiens* | *R. norvegicus* | *M. musculus* | *X. laevis* | B8JIY2 | Q7ZUM1 |
| *H. sapiens* | - | 74 | 79 | 57 | 57 | 59 |
| *R. norvegicus* | 74 | - | 90 | 50 | 57 | 60 |
| *M. musculus* | 79 | 90 | - | 67 | 68 | 62 |
| *X. laevis* | 57 | 50 | 67 | - | 60 | 63 |
| B8JIY2 | 57 | 57 | 68 | 60 | - | 100 |
| Q7ZUM1 | 59 | 60 | 62 | 63 | 100 | - |
